# Impact of modified albumin–bilirubin grade on survival in patients with HCC who received lenvatinib

**DOI:** 10.1038/s41598-021-93794-5

**Published:** 2021-07-14

**Authors:** Toshifumi Tada, Takashi Kumada, Atsushi Hiraoka, Masanori Atsukawa, Masashi Hirooka, Kunihiko Tsuji, Toru Ishikawa, Koichi Takaguchi, Kazuya Kariyama, Ei Itobayashi, Kazuto Tajiri, Noritomo Shimada, Hiroshi Shibata, Hironori Ochi, Satoshi Yasuda, Hidenori Toyoda, Shinya Fukunishi, Hideko Ohama, Kazuhito Kawata, Joji Tani, Shinichiro Nakamura, Kazuhiro Nouso, Akemi Tsutsui, Takuya Nagano, Tanaka Takaaki, Norio Itokawa, Tomomi Okubo, Taeang Arai, Michitaka Imai, Kouji Joko, Yohei Koizumi, Yoichi Hiasa, Real-life Practice Experts for HCC (RELPEC) Study Group and the HCC 48 Group (hepatocellular carcinoma experts from 48 clinics in Japan)

**Affiliations:** 1 Department of Internal Medicine, Japanese Red Cross Himeji Hospital, 1-12-1 Shimoteno, Himeji, Hyogo 670-8540 Japan; 2 Faculty of Nursing, Gifu Kyoritsu University, Ogaki, Japan; 3 Gastroenterology Center, Ehime Prefectural Central Hospital, Matsuyama, Japan; 4 Division of Gastroenterology and Hepatology, Department of Internal Medicine, Nippon Medical School, Tokyo, Japan; 5 Department of Gastroenterology and Metabology, Ehime University Graduate School of Medicine, Matsuyama, Japan; 6 Center of Gastroenterology, Teine Keijinkai Hospital, Sapporo, Japan; 7 Department of Gastroenterology, Saiseikai Niigata Hospital, Niigata, Japan; 8 Department of Hepatology, Kagawa Prefectural Central Hospital, Takamatsu, Japan; 9 Department of Gastroenterology, Okayama City Hospital, Okayama, Japan; 10 Department of Gastroenterology, Asahi General Hospital, Asahi, Japan; 11 Department of Gastroenterology, Toyama University Hospital, Toyama, Japan; 12 Division of Gastroenterology and Hepatology, Otakanomori Hospital, Kashiwa, Japan; 13 Department of Gastroenterology, Tokushima Prefectural Central Hospital, Tokushima, Japan; 14 Hepato-Biliary Center, Matsuyama Red Cross Hospital, Matsuyama, Japan; 15 Department of Gastroenterology and Hepatology, Ogaki Municipal Hospital, Ogaki, Japan; 16 Second Department of Internal Medicine, Osaka Medical College, Takatsuki, Japan; 17 Hepatology Division, Department of Internal Medicine, Hamamatsu University School of Medicine, Hamamatsu, Japan; 18 Department of Gastroenterology and Neurology, Kagawa University School of Medicine, Miki, Kagawa Japan

**Keywords:** Gastroenterology, Hepatology

## Abstract

We investigated the impact on survival of modified albumin–bilirubin (mALBI) grade versus Child–Pugh classification in patients with hepatocellular carcinoma (HCC) who received lenvatinib. A total of 524 patients with HCC who received lenvatinib were included. Univariate analysis showed that mALBI grade 2b/3 and Child–Pugh class B/C were significantly associated with survival [hazard ratio (HR), 2.471; 95% confidence interval (CI), 1.944–3.141 and HR, 2.178; 95%CI, 1.591–2.982]. In patients with a Child–Pugh score of 5, multivariate analysis showed that mALBI grade 2b/3 was independently associated with survival (HR, 1.814; 95%CI, 1.083–3.037). Conversely, among patients with mALBI grade 1/2a, there was no difference in survival between those with a Child–Pugh class of 5 or 6 (*p* = 0.735). Time-dependent receiver operating characteristic analysis showed that the ALBI score predicted survival better than the Child–Pugh score. The optimal cut-off value of the ALBI score for predicting survival was nearly the same as the value separating mALBI grades 2a and 2b. In conclusion, the mALBI grade was a better predictor of survival than the Child–Pugh classification in patients with unresectable HCC who received lenvatinib therapy.

## Introduction

Hepatocellular carcinoma (HCC) accounts for the majority of malignancy in the liver. It is also one of the most prevalent cancers worldwide and the cause of important health-related problems, making it the third most frequent cause of cancer-related deaths in the world^1–3^. Curative treatments such as hepatic resection, radiofrequency/microwave ablation, and transplantation are performed in only 30–40% of patients with HCC. Remaining patients for whom curative treatment is not indicated undergo transarterial chemoembolization or systemic drug therapy for palliation^4^.

Sorafenib^5,6^, regorafenib^7,8^, and ramucirumab^9^ are attributed as molecularly targeted agents and have been developed for the systemic drug therapy of patients with unresectable HCC. In the past 10 years, no first-line systemic drug therapies other than sorafenib were approved for patients with unresectable HCC in Japan^5–8^. Lenvatinib^10^, a newly developed tyrosine kinase inhibitor, has recently become available not only as a first-line therapy in patients with unresectable HCC, but also as a later-line therapeutic option^11,12^. More recently in Japan, the combination of atezolizumab plus bevacizumab has been approved as a first-line systemic therapy for patients with unresectable HCC^13^.

The Child–Pugh classification system^14^ is the most extensively and globally used method for evaluating hepatic function in patients being treated for HCC. However, this system includes subjective factors such as hepatic encephalopathy and ascites, and interrelated factors such as serum albumin and ascites. Therefore, accurate and more objective clinical markers to evaluate hepatic function are desired. A new method for assessing hepatic function, known as the albumin–bilirubin (ALBI) grade, was recently developed^15^. In addition, several studies have shown that the modified ALBI (mALBI) grade is effective for assessing hepatic function in patients with HCC^16–18^. Furthermore, we recently reported that hepatic function in patients with naïve HCC has improved remarkably throughout 30 years, and in the last 5 years over 80% of patients reached Child–Pugh class A, including 65% who attained a Child–Pugh score of 5^19^. In addition, we recently reported that Child–Pugh score of 6 was independently associated with overall survival in HCC patients with Child–Pugh class A who were treated with sorafenib [hazard ratio (HR), 1.41; 95% confidence interval (CI), 1.08–1.84]^20^. Since lenvatinib therapy is mainly indicated in HCC patients with Child–Pugh class A disease, it is necessary to elucidate the ability of the mALBI grade to assess hepatic function as an alternative to the Child–Pugh classification system.

In this study, we investigated the association between the ALBI score and overall survival, with a particular focus on the mALBI grade versus the Child–Pugh score, in patients with unresectable HCC who treated with lenvatinib at multiple centers in Japan. To further compare the ability of the ALBI score and Child–Pugh score in predicting overall survival, we generated time-dependent receiver operating characteristic (ROC) curves analysis^21^ for censored data and compared the areas under the ROC curves (AUROCs).

## Results

### Patient characteristics

Table 1 shows the characteristics of the study patients at the baseline. There were 126 (24.0%) females and 398 (76.0%) males, with a median age of 73.0 (68.0–79.0) years. The median follow-up period was 11.6 (6.0–18.5) months. There were 277 (52.9%) patients with a Child–Pugh score of 5, 171 (32.6%) with a score of 6, 56 (10.7%) with a score of 7, 15 (2.9%) with a score of 8, 2 (0.4.%) with a score of 9, 2 (0.4%) with a score of 10, and 1 (0.2%) with a score of 13, corresponding to 448 (85.5%) patients with Child–Pugh class A disease, 73 (13.9%) with class B disease, and 3 (0.6%) patients with class C disease. The median ALBI score was − 2.36 (− 2.68 to − 1.99). There were 165 (31.5%) patients with mALBI grade 1, 131 (25.0%) with grade 2a, 210 (40.1%) with grade 2b, and 18 (3.4%) with grade 3.

**Table 1:** Patient characteristics.

	Overall (n = 524)	Child–Pugh A (n = 448)	Child–Pugh score of 5 (n = 277)
Age (years)*	73.0 (68.0–79.0)	73.0 (68.0–79.0)	73.0 (68.0–79.0)
Sex (female/male)	126/398	106/342	59/218
ECOG-PS (0/1/2/3)	420/91/12/1	367/72/8/1	233/40/3/1
Body mass index (kg/m^2^)*	22.9 (20.6–25.4)	22.9 (20.8–25.5)	23.1 (20.9–25.4)
Etiology of HCC (hepatitis B/C/B + C/non-B, non-C)	75/214/2/233	69/179/2/198	49/105/2/121
Albumin (g/dL)*	3.6 (3.3–4.0)	3.8 (3.4–4.0)	3.9 (3.7–4.2)
Total bilirubin (mg/dL)*	0.8 (0.6–1.1)	0.7 (0.6–1.0)	0.7 (0.6–1.0)
Platelet count (× 10^3^/m^3^)*	13.5 (10.0–18.6)	13.5 (10.2–18.1)	14.3 (10.8–18.5)
Prothrombin time (%)*	87.0 (78.0–97.0)	88.3 (80.0–98.0)	90.0 (83.0–100.0)
α-fetoprotein (ng/mL)*	40.9 (6.8–668.7)	35.0 (6.1–497.2)	25.7 (5.6–426.0)
ALBI score*	− 2.36 (− 2.68 to − 1.99)	− 2.47 (− 2.73 to − 2.17)	− 2.65 (− 2.86 to − 2.44)
mALBI grade (1/2a/2b/3)	165/131/210/18	165/130/152/1	153/95/28/1
Child–Pugh score (5/6/7/8/9/10/13)	277/171/56/15/2/2/1	277/171	
Child–Pugh class (A/B/C)	448/73/3		
BCLC stage (0/A/B/C/D)	4/8/225/285/2	4/8/198/237/1	3/7/126/140/1
Vascular invasion (yes/no)	116/408	95/353	51/226
Extrahepatic spread (yes/no)	186/338	158/290	96/181
Molecular targeted therapy experience (yes/no)	155/369	133/315	79/198
Line of lenvatinib (1st/2nd/3rd/4th)	369/110/44/1	315/95/37/1	198/59/20/0
Post-treatment of lenvatinib (yes/no/ongoing)	188/253/83	169/204/75	125/101/51
Follow-up duration (months)*	11.6 (6.0–18.5)	12.4 (7.0–19.2)	14.3 (10.8–18.5)

*Values are expressed as medians (interquartile range).

*ECOG-PS* Eastern Cooperative Oncology Group performance status, *HCC* Hepatocellular carcinoma, *ALBI* Albumin–bilirubin, *mALBI* Modified albumin–bilirubin, *BCLC* Barcelona clinic liver cancer.

The cumulative overall survival rates at 6, 12, 18, and 24 months were 82.7%, 61.6%, 47.3%, and 37.0%, respectively. The median overall survival was 17.1 months (95% CI, 15.0–19.6). There were 45 (27.3%), 44 (33.6%), 59 (28.1%), and 7 (38.9%) patients with mALBI grade 1/2a/2b/3 who had an experience of molecular targeted therapy (*p* = 0.493). Additionally, there were 133 (29.7%), 21 (28.8%), and 1 (33.3%) patients with Child–Pugh class A/B/C who had an experience of molecular targeted therapy (*p* = 0.977).

### Overall survival by mALBI grade and Child–Pugh class

**Figure 1:**
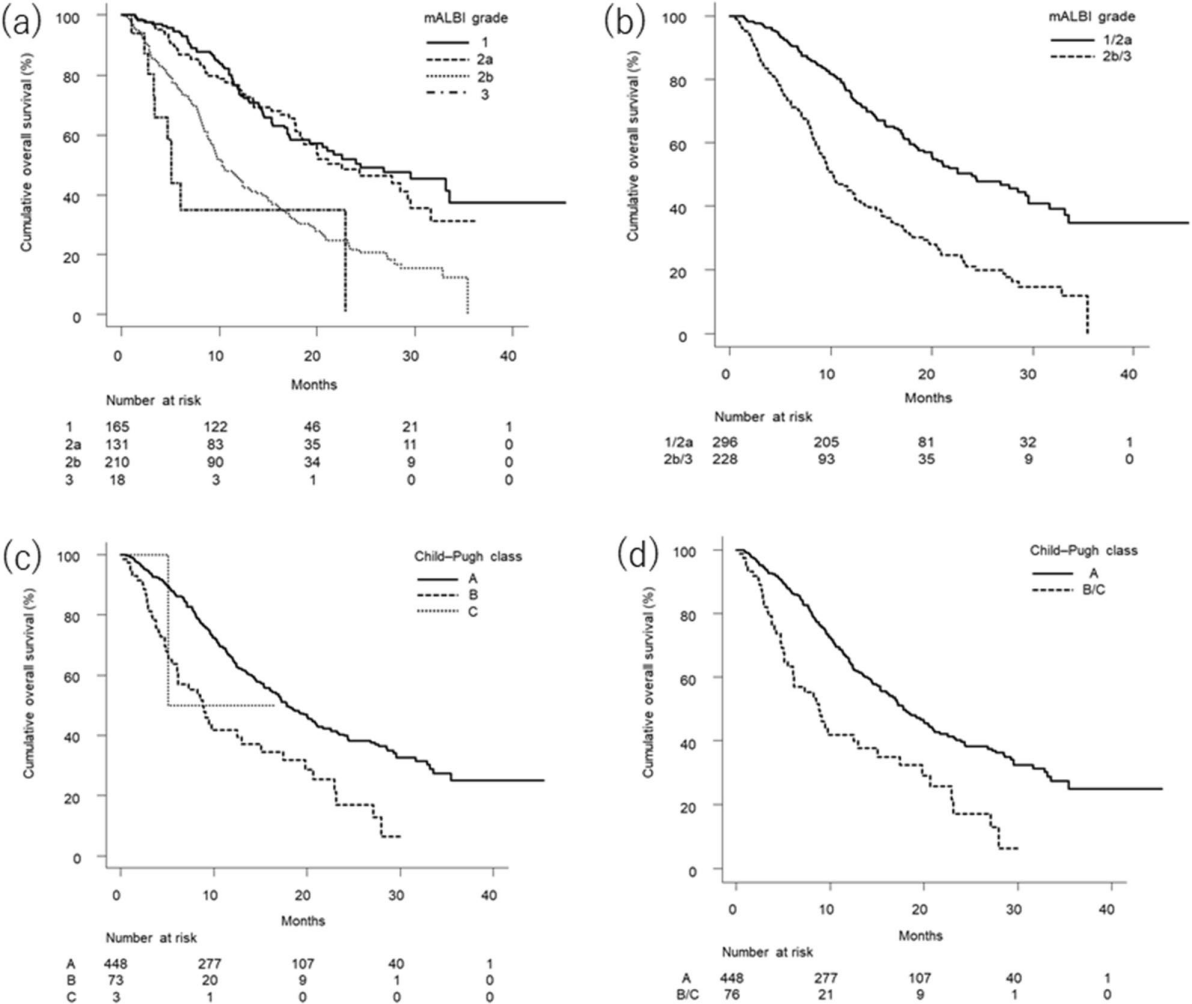
Cumulative survival curves for all study patients. (**a**) By mALBI grade. There was a significant difference in cumulative overall survival among patients grouped by mALBI grade (*p* < 0.001). The median survival times of patients with mALBI grades 1, 2a, 2b, and 3 were 24.4 (95%CI, 17.2—not available), 22.5 (95%CI, 18.3–29.5), 10.3 (95%CI, 9.1–12.5), and 5.1 (95%CI, 3.3—not available) months, respectively. Multiple comparisons between mALBI grades 1 and 2a, 1 and 2b, 1 and 3, 2a and 2b, 2a and 3, and 2b and 3 demonstrated p values of 1.000, < 0.001, < 0.001, < 0.001, < 0.001, and 0.654, respectively. (**b**) By mALBI grade divided into 1/2a and 2b/3. There was a significant difference in cumulative overall survival between patients with mALBI grades 1/2a and 2b/3 (*p* < 0.001). The median survival times in patients with mALBI grades 1/2a and 2b/3 were 23.9 (95%CI, 20.0–29.5) and 10.1 (95%CI, 8.9–12.4) months, respectively. (**c**) By Child–Pugh classification. There was a significant difference in cumulative overall survival among patients stratified by Child–Pugh classification (*p* < 0.001). The median survival times in patients with Child–Pugh classes A, B, and C were 17.8 (95%CI, 16.0–20.8), 8.8 (95%CI, 6.0–12.9), and 5.1 (95%CI, 5.1—not available) months, respectively. Multiple comparisons between Child–Pugh classes A and B, A and C, and B and C demonstrated p values of < 0.001, 1.000, and 1.000, respectively. (**d**) By Child–Pugh classification divided into A and B/C. There was a significant difference in cumulative overall survival between patients with Child–Pugh classes A and B/C (*p* < 0.001). The median survival times in patients with Child–Pugh classes A and B/C were 17.8 (95%CI, 16.0–20.8) and 8.8 (95%CI, 6.0–15.0) months, respectively. *mALBI* Modified albumin–bilirubin, *CI* Confidence interval.

Figure 1a shows that cumulative overall survival curves differed significantly according to mALBI grade (*p* < 0.001). Multiple comparisons using the Bonferroni method showed significant differences between each grade, except between mALBI grades 1 and 2a, and grades 2b and 3. Figure 1b shows a significant difference between the cumulative overall survival curves of patients with mALBI grades of 1/2a versus 2b/3 (*p* < 0.001). Univariate Cox proportional hazards model analysis showed that mALBI grade 2b/3 was significantly associated with poor overall survival (HR, 2.471; 95%CI, 1.944–3.141; *p* < 0.001; c-index, 0.625). Multivariate Cox proportional hazards models analysis that included the covariates of age, sex, ECOG-PS, etiology, α-fetoprotein, HCC stage, and mALBI grade showed that HCC stage (HR, 1.412) and mALBI grade (HR, 2.399) were independently associated with overall survival (Supplementary Table 1).

Figure 1c shows that cumulative overall survival curves differed significantly according to Child–Pugh class (*p* < 0.001). Multiple comparisons using the Bonferroni method showed a significant difference only between Child–Pugh classes A and B. Figure 1d shows a significant difference between cumulative overall survival curves for Child–Pugh class A versus B/C (*p* < 0.001). Univariate Cox proportional hazards model analysis showed that Child–Pugh class B/C was significantly associated with poor overall survival (HR, 2.178; 95%CI, 1.591–2.982; *p* < 0.001; c-index, 0.555). Multivariate Cox proportional hazards models analysis that included the covariates of age, sex, ECOG-PS, etiology, α-fetoprotein, HCC stage, and Child–Pugh class showed that HCC stage (HR, 1.402) and Child–Pugh class (HR, 2.048) were independently associated with overall survival (Supplementary Table 2).

### Therapeutic response

Radiological best response rates according to the mALBI grade and Child–Pugh class show supplementary Tables 3 and 4, respectively. Overall response rate (ORR) and disease control rate (DCR) in patients with mALBI grade 1/2a and 2b/3 were 40.8% and 83.0%, and 32.3% and 75.3%, respectively. DCR was significantly difference between the patients with mALBI grade 1/2a and 2b/3 (*p* = 0.049) (Supplementary Table 3). In addition, ORR and DCR in patients with Child–Pugh class A and B/C were 38.6% and 82.6%, and 28.8% and 62.1%, respectively. DCR was significantly difference between the patients with Child–Pugh class A and B/C (*p* < 0.001) (Supplementary Table 4).

### Adverse events

Supplementary Tables 5 and 6 lists the treatment-related adverse events that occurred in study patients according to the mALBI grade and Child–Pugh class. Regarding to the relationship between adverse events, liver function, and overall survival, multivariate Cox proportional hazards models analysis that included the covariates of adverse events and mALBI grade showed that palmar-plantar erythrodysesthesia (HR, 0.679), proteinuria (HR, 0.646), and mALBI grade (HR, 2.234) were independently associated with overall survival (Supplementary Table 7). In addition, multivariate Cox proportional hazards models analysis that included the covariates of adverse events and Child–Pugh class showed that palmar-plantar erythrodysesthesia (HR, 0.648), proteinuria (HR, 0.584), and Child–Pugh class (HR, 2.021) were independently associated with overall survival (Supplementary Table 8).

### Subgroup analysis

**Figure 2:**
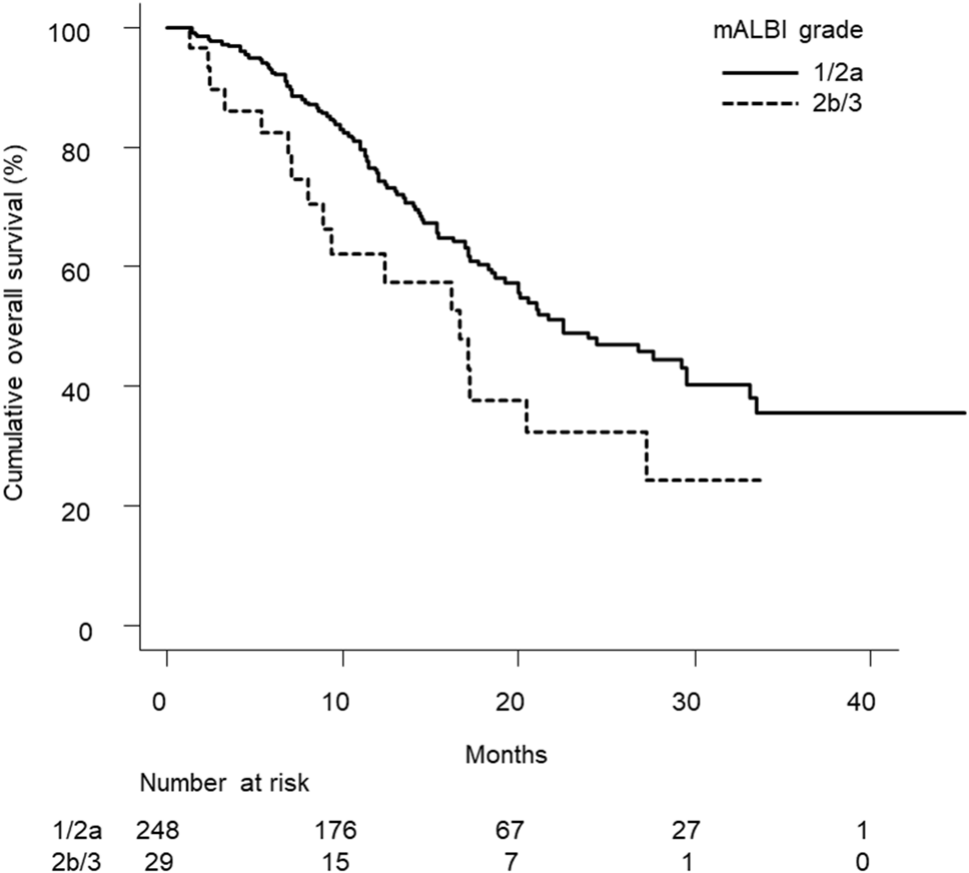
Cumulative survival curves in patients with Child–Pugh score of 5. There was a significant difference in cumulative overall survival between patients with mALBI grades 1/2a and 2b/3 (*p* = 0.032). The median survival times in patients with mALBI grades 1/2a and 2b/3 were 22.5 (95%CI, 19.2–29.5) and 16.6 (95%CI, 8.0–27.3) months, respectively.

Table 1 shows the characteristics of patients with a Child–Pugh score of 5 at the start of follow-up (n = 277). There were 59 females and 218 males, with a median age of 73.0 (68.0–79.0) years. There were 153 (55.2%) patients with mALBI grade 1, 95 (34.3%) with grade 2a, 28 (10.1%) with grade 2b, and 1 (0.4%) with grade 3. Figure 2 shows a significant difference between cumulative overall survival curves for mALBI grade 1/2a versus 2b/3 (*p* = 0.032). Univariate Cox proportional hazards model analysis showed that mALBI grade 2b/3 was significantly associated with poor overall survival (HR, 1.740; 95%CI, 1.040–2.909; *p* = 0.035). Multivariate Cox proportional hazards models analysis that included the covariates of age, sex, ECOG-PS, etiology, α-fetoprotein, HCC stage, and mALBI grade showed that mALBI grade was independently associated with overall survival (HR, 1.814; 95%CI, 1.083–3.037; *p* = 0.024) (Table 2). There were 116 (58.0%) and 84 (42.0%) patients with mALBI grade 1/2a who received post-treatment of lenvatinib and not, and 9 (34.6%) and 17 (65.4%) patients with mALBI grade 2b/3 who received post-treatment of it and not, respectively (*p* = 0.035).

**Table 2:** Multivariate analysis in patients with Child–Pugh score of 5.

	HR	95% CI	*p* value
**Age (years)**			
< 75 (n = 161)	1	0.899–1.945	0.155
≥ 75 (n = 116)	1.322		
**Sex**			
Female (n = 59)	1	0.675–1.680	0.786
Male (n = 218)	1.065		
**ECOG-PS**			
0 (n = 233)	1	0.506–1.562	0.682
≥ 1 (n = 44)	0.889		
**Etiology of HCC**			
Viral (n = 156)	1	0.497–1.079	0.115
Non-viral (n = 121)	0.732		
**α-fetoprotein (ng/mL)**			
< 400 (n = 205)	1	0.938–2.120	0.099
≥ 400 (n = 71)	1.410		
**mALBI grade**			
1/2a (n = 248)	1	1.083–3.037	0.024
2b/3 (n = 29)	1.814		
**BCLC stage**			
≤ B (n = 136)	1	0.969–2.209	0.070
≥ C (n = 141)	1.463		

*HR* Hazard ratio, *CI* Confidence interval, *ECOG-PS* Eastern Cooperative Oncology Group performance status, *HCC* Hepatocellular carcinoma, *mALBI* Modified albumin–bilirubin, *BCLC* Barcelona clinic liver cancer.

**Figure 3:**
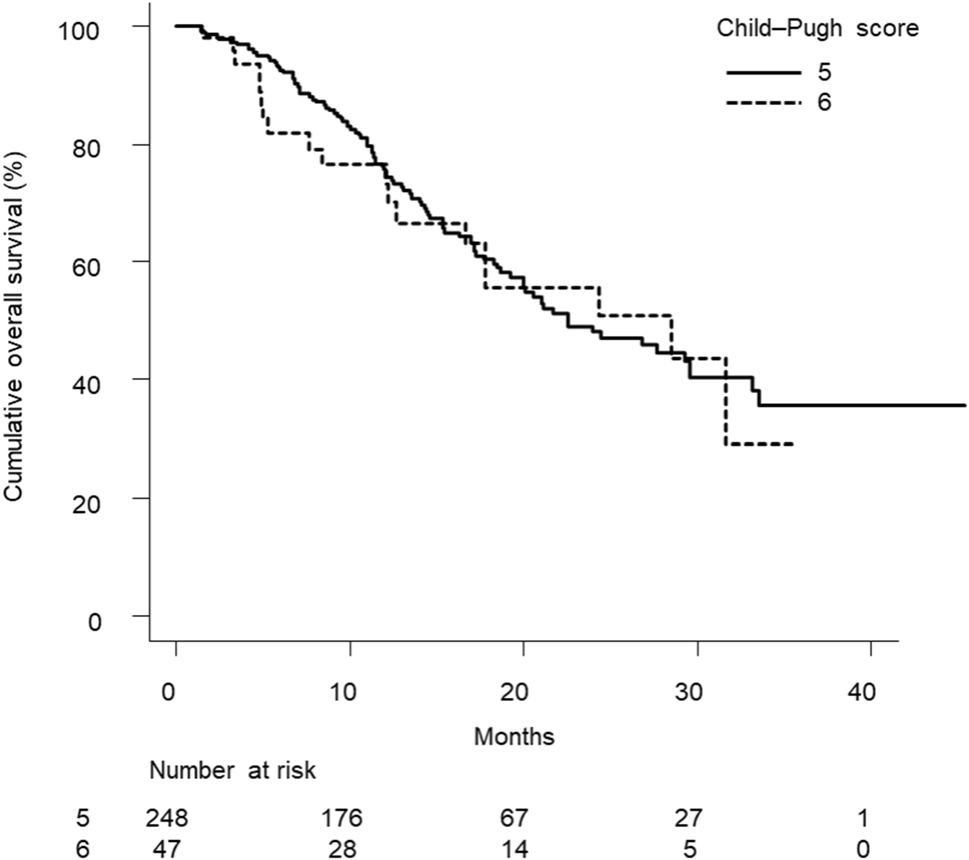
Cumulative survival curves in patients with mALBI grade 1/2a and Child–Pugh class A. There was no difference in cumulative overall survival between patients with mALBI grade 1/2a and Child–Pugh classes 5 and 6 (*p* = 0.735). The median survival times in patients with mALBI grade 1/2a and Child–Pugh classes 5 and 6 were 22.5 (95%CI, 19.2–29.5) and 28.5 (95%CI, 12.7—not available) months, respectively. *mALBI* Modified albumin–bilirubin.

Figure 3 shows that in patients with mALBI grade 1/2a (n = 295), there was no significant difference in cumulative overall survival curves according to Child–Pugh scores of 5 versus 6 (*p* = 0.735). Univariate Cox proportional hazards model analysis showed that a Child–Pugh class of 6 was not associated with poor overall survival (HR, 1.088; 95%CI, 0.666–1.777; *p* = 0.736).

### Time-dependent ROC analysis of overall survival

Supplementary Fig. 1a–h shows the ROC curves of ALBI scores for overall survival at 3, 6, 9, 12, 15, 18, 21, and 24 months, respectively, after the start of follow-up using time-dependent ROC analysis. The AUCs at 3, 6, 9, 12, 15, 18, 21, and 24 months were 0.682, 0.693, 0.673, 0.640, 0.641, 0.621, 0.615, and 0.621, respectively. Supplementary Fig. 2a–h shows the ROC curves of Child–Pugh scores for overall survival at 3, 6, 9, 12, 15, 18, 21, and 24 months, respectively, after the start of follow-up using time-dependent ROC analysis. The AUCs at 3, 6, 9, 12, 15, 18, 21, and 24 months were 0.738, 0.719, 0.712, 0.673, 0.663, 0.658, 0.664, and 0.671, respectively.

**Figure 4:**
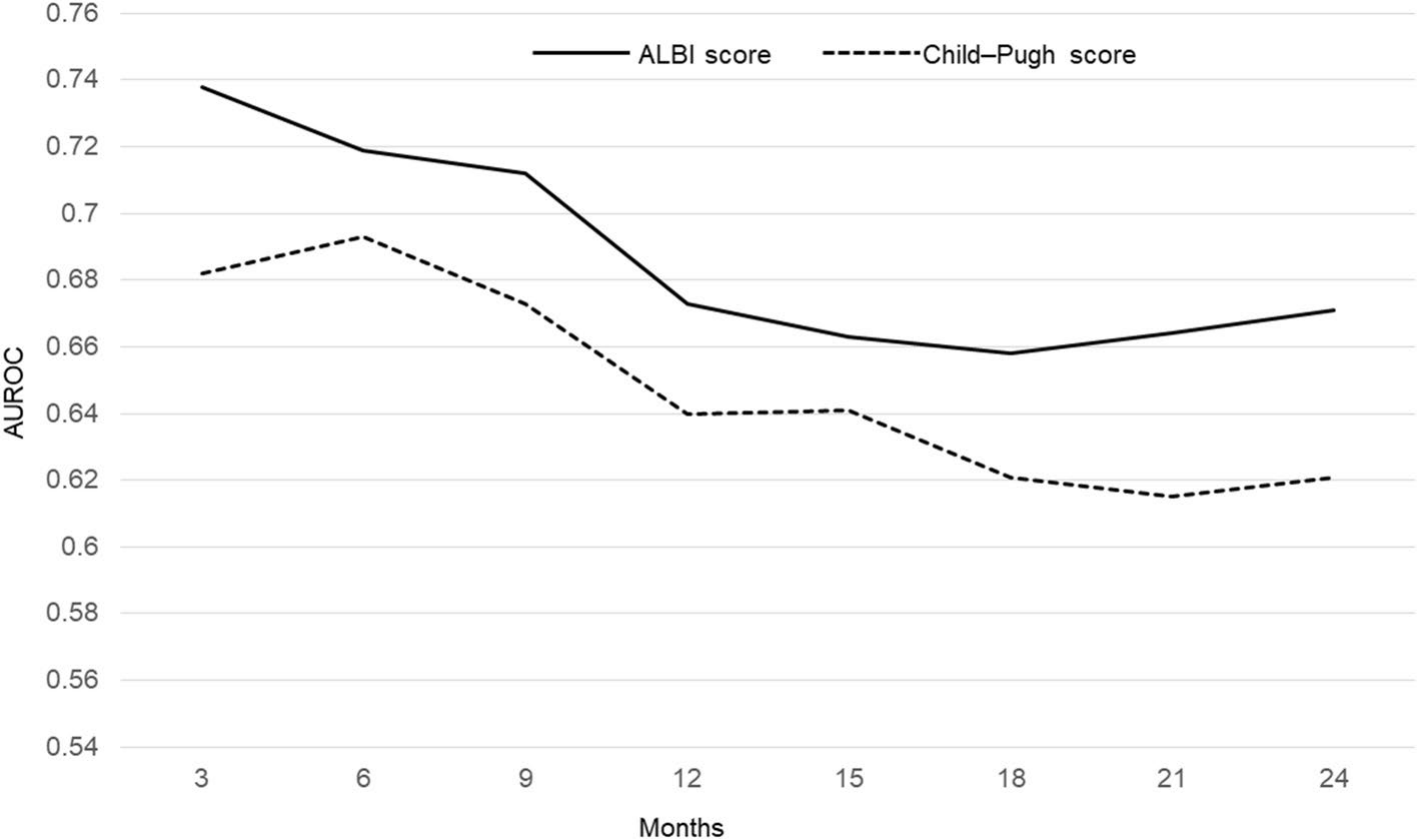
Time-dependent AUROCs of the ALBI and Child–Pugh scores for overall survival after the start of follow-up. Based on time-dependent ROC analysis, the AUROCs of the ALBI and Child–Pugh scores for overall survival at 3, 6, 9, 12, 15, 18, 21, and 24 months were 0.738 and 0.682, 0.719 and 0.693, 0.712 and 0.673, 0.673 and 0.640, 0.663 and 0.641, 0.658 and 0.621, 0.664 and 0.615, and 0.671 and 0.621, respectively. Based on time-dependent AUROCs, the ALBI score had a higher predictive power for overall survival than the Child–Pugh score. *AUROC* Area under the receiver operating characteristic curve, *ALBI* Albumin–bilirubin.

Figure 4 shows AUROC plots of the ALBI and Child–Pugh scores for overall survival from 3 to 24 months after the start of follow-up, as analyzed by time-dependent ROC. The ALBI score had better predictive ability for overall survival than the Child–Pugh score at all time points.

Table 3 shows the sensitivity, specificity, and cut-off values of the ALBI and Child–Pugh scores at 6, 12, 18, and 24 months according to time-dependent ROC analysis for overall survival. The optimal cut-off values of the ALBI score for predictive overall survival at each month were nearly identical to the value that separated mALBI grades 2a and 2b. The optimal cut-off value of the Child–Pugh score for predictive overall survival was 5 at each month.

**Table 3:** Sensitivity, specificity, and cut-off values based on time-dependent ROC analysis.

	ALBI score			Child–Pugh score		
	Cut-off value	Sensitivity (%)	Specificity (%)	Cut-off value	Sensitivity (%)	Specificity (%)
At 6 months	− 2.19	70.0	67.8	5	73.9	58.5
At 12 months	− 2.24	61.3	71.9	5	63.1	62.8
At 18 months	− 2.24	54.4	74.3	5	58.1	65.1
At 24 months	− 2.31	56.8	73.9	5	55.2	66.6

*ROC* Receiver operating characteristic.

## Discussion

In this multicenter study of a large number of patients with unresectable HCC who received lenvatinib therapy, Cox proportional hazards modeling that included age, sex, ECOG-PS, HCC etiology, mALBI grade, α-fetoprotein, and BCLC stage as covariates showed that mALBI grade (1/2a vs. 2b/3) was independently associated with overall survival in patients with a Child–Pugh score of 5. Conversely, the survival analysis of patients with mALBI grade 1/2a and Child–Pugh class A showed no difference between patients with Child–Pugh class 5 versus 6. In addition, the predictive power of the ALBI score for overall survival was superior to that of the Child–Pugh score in time-dependent ROC analysis. Furthermore, the optimal cut-off values of the ALBI score for predicting good survival in the 2 years from the start of lenvatinib treatment were nearly equal to the value separating mALBI grades 2a and 2b. These results suggest that the ALBI score is a better predictive marker for overall survival than the Child–Pugh score in patients with unresectable HCC who are treated with lenvatinib. In addition, the mALBI grade was able to predict good versus poor prognosis in patients with a Child–Pugh score of 5, even though these patients are generally considered to have a good prognosis. Furthermore, patients with a mALBI grade of 1 or 2a had a similar prognosis regardless of whether their Child–Pugh score was 5 or 6. In the present study, the median overall survival was longer than those of RFLECT trial^10^ (17.1 vs. 13.6 months). It was considered that the evolution of post-treatment of lenvatinib and the good management of adverse events were influenced the improvement of median overall survival.

Recently, Ueshima et al.^22^ investigated the association between baseline liver function, as determined by the Child–Pugh score and ALBI grade, and the outcomes of 82 patients with unresectable HCC who were treated with lenvatinib. Their study patients were divided into four groups: (1) Child–Pugh score 5 and ALBI grade 1 (group 1; n = 27); (2) Child–Pugh score 5 and ALBI grade 2 (group 2; n = 19); (3) Child–Pugh score 6 (group 3; n = 30); and (4) Child–Pugh score ≥ 7 (group 4; n = 6). They found that the median times to treatment failure (i.e., time from the initial administration of lenvatinib to treatment discontinuation for any reason, including disease progression, treatment toxicity, patient preference, and any cause of death) were 8.9, 5.3, 5.9 and 0.3 months in groups 1, 2, 3, and 4, respectively (HR, 0.38; 95%CI, 0.18–0.80; *p* < 0.001)^22^. In addition, overall survival was significantly better in patients with ALBI grade 1 than in those with ALBI grade 2 (HR, 0.12; 95%CI, 0.02–0.97); *p* < 0.01)^22^. Very recently, Tsuchiya et al.^23^ investigated the factors associated with overall survival in 343 patients with unresectable HCC who were treated with lenvatinib. They found that ECOG-PS ≥ 1 (HR, 1.50; 95%CI, 1.09–2.08; *p* = 0.014), mALBI grade 2b/3 (HR, 1.56; 95%CI, 1.09–2.17; *p* = 0.012), α-fetoprotein ≥ 400 (HR, 2.00; 95%CI, 1.42–2.80; *p* < 0.001), major vascular invasion (HR, 1.91; 95%CI, 1.26–2.89; *p* = 0.002), and molecular targeted therapy experience (HR, 2.22; 95%CI, 1.56–3.13; *p* < 0.001) were independently associated with overall survival in the multivariate analysis. Although our study did not investigate the time to treatment failure, we showed that patients with mALBI grade 1, as well as those with 2a, had better overall survival than patients with mALBI grade 2b/3. In addition, we used time-dependent ROC analysis to demonstrate that the optimal cut-off values of the ALBI score during the 2 years after initiating lenvatinib treatment were nearly equal to the value that separated mALBI grades 2a and 2b, as opposed to that separating ALBI grades 1 and 2. One advantage of this study relative to that by Ueshima et al. and Tsuchiya et al. is that the former included more patients with unresectable HCC who received lenvatinib therapy. In addition, this study statistically confirmed the optimal cut-off values of the ALBI score for predicting good overall survival in patients treated with lenvatinib.

Ando et al.^24^ reported that mALBI grade 1/2a (odds ratio, 5.18; 95%CI, 1.465–18.31; *p* = 0.011) was an independent factor for possible treatment with second-line molecularly targeted agents in 141 HCC patients who received lenvatinib as first-line therapy. In addition, in a study by Hiraoka et al.^25^ using a Japanese hospital-based administration database, the overall duration of systematic treatment in patients with advanced HCC was shorter in those with a baseline ALBI grade of 2b or 3 than in those with grade 1 or 2a (medians: 7.1, 6.7, 4.5, and 3.0 months for grades 1, 2a, 2b, and 3, respectively). In this study, we clarified that there was a significant difference in the post-treatment of lenvatinib rate between patients with mALBI grade 1/2a and those with grade 2b/3, even among those with good liver function as defined by a Child–Pugh score of 5.

It is well known that immunology regulations may affect HCC progression and it was reported that immunodeficiency may promote adaptive alterations of host gut- or tissue-based microbiome^26^. Therefore, it was considered that these phenomena may be influenced mALBI grade as potential mechanisms and associated with survival in patients with HCC who received lenvatinib.

The mRNA modifications are potentially new insights into this biological basis, especially N4-acetylcytidine on RNA expression^27^. The recent progress in N4-acetylcytidine on RNA expression is also playing key role on the development of HCC and survival in patients with HCC. Further prospective studies with considering the relationship between mALBI grade and mRNA modifications/ N4-acetylcytidine, including additional potential mechanisms, in patients with HCC are warranted.

The Child–Pugh score/classification system comprises five factors, specifically serum albumin, total bilirubin, prothrombin time, ascites, and encephalopathy^14^. This system has been widely used to evaluate hepatic function and has been incorporated into the HCC staging system^28^. However, the Child–Pugh score is limited by the subjectivity involved in evaluating hepatic encephalopathy and ascites, and serum albumin levels are associated with the severity of ascites^14^. Furthermore, this hepatic function assessment system was originally developed for patients with cirrhosis, not HCC. The ALBI grade, which was recently developed as an objective biomarker for assessment of hepatic function, is calculated using only serum albumin and total bilirubin levels. It has been shown to accurately predict the prognosis of patients with HCC, and is superior not only to the Child–Pugh classification^20,29^, but also to the liver damage classification system^30^.

Hiraoka et al.^16^ developed the new mALBI grading system, which divides ALBI grade 2 into 2a and 2b, by analyzing 46,681 HCC patients in a nationwide survey conducted in Japan. The mALBI grade showed a good ability to stratify prognosis in each TNM stage of the Liver Cancer Study Group of Japan^31^, and there was a statistically significant difference between each mALBI grade in all TMN stages (*p* < 0.01). This study confirmed the utility of subdividing ALBI grade 2 into 2a and 2b for predicting survival in advanced HCC patients who received systemic therapy.

ROC analysis is generally used to assess the discriminatory ability of a continuous variable for a binary disease outcome (e.g., disease positive or negative). However, majority of chronic disease outcomes, including the prognosis of patients with cancer, are time dependent. Therefore, time-dependent ROC curve analysis has been used to assess the predictive ability of clinical markers for time-dependent disease outcomes^21^. No previous studies have used this statistical method to assess parameters of hepatic function regarding to their association with overall survival in patients with advanced HCC who treated with lenvatinib. In this study, the AUROCs by time-dependent ROC curve analysis showed that the ALBI score was superior to the Child–Pugh score in terms of predicting overall survival up to 2 years after the start of lenvatinib therapy in patients with unresectable HCC.

The main limitations of this study are its hospital-based subject population and retrospective nature. Although the study included a large number of patients with unresectable HCC from multiple centers in Japan, future prospective studies with community-based populations are warranted. An additional limitation was that the study only enrolled patients with unresectable HCC who were treated with lenvatinib. Future studies of patients with HCC should include those who receive atezolizumab plus bevacizumab, a recently approved, first-line, systemic combination therapy, as well as those who receive lenvatinib.

In conclusion, the mALBI grade is a new, simple, objective parameter that was a better predictor of survival in HCC patients who received lenvatinib therapy than the Child–Pugh classification, even among individuals with good liver function as defined by a Child–Pugh score of 5. Further studies in other populations are warranted to confirm these findings.

## Methods

### Patients

The protocol used in the present study was approved by the Institutional Ethics Committee of Ehime Prefectural Central Hospital (IRB No. 30–66), based on the Guidelines for Clinical Research issued by the Ministry of Health and Welfare of Japan. All methods were carried out in accordance with relevant guidelines and regulations.

We enrolled 524 patients with unresectable HCC who received lenvatinib between March 2018 and February 2021 at 19 institutions in Japan [Himeji Red Cross Hospital (n = 126), Nippon Medical School Hospital Group (Sendagi Hospital, Chiba Hokusoh Hospital, and Musashi Kosugi Hospital) (n = 75), Ehime Prefectural Central Hospital (n = 53), Ogaki Municipal Hospital (n = 38), Kagawa University Hospital (n = 33), Asahi General Hospital (n = 26), Ehime University Hospital (n = 23), Osaka Medical College Hospital (n = 22), Okayama City Hospital (n = 21), Teine Keijinkai Hospital (n = 20), Saiseikai Niigata Hospital (n = 18), Kagawa Prefectural Central Hospital (n = 17), Hamamatsu University Hospital (n = 17), Matsuyama Red Cross Hospital (n = 16), Otakanomori Hospital (n = 9), Toyama University Hospital (n = 6), and Tokushima Prefectural Central Hospital (n = 4)].

The start of the follow-up period was defined as the date when lenvatinib therapy began. The end of follow-up was defined as the date of the final visit for patients who remained alive or the date of death for patients who died during the follow-up period.

The etiology of HCC was considered to be hepatitis B virus in patients positive for hepatitis B virus surface antigen, and hepatitis C virus in those positive for hepatitis C virus antibodies.

### Diagnosis and treatment of HCC

HCC was diagnosed according to increases in α-fetoprotein levels, pathological findings, or the use of imaging modalities such as dynamic computed tomography, gadolinium ethoxybenzyl diethylenetriamine pentaacetic acid-enhanced magnetic resonance imaging, and contrast-enhanced ultrasonography with perflubutane^32,33^. HCC stage was based on the Barcelona Clinic Liver Cancer (BCLC) classification system^28^.

The most appropriate treatment modality for HCC in each patient was selected through discussion between surgeons, hepatologists, and radiologists in each institution, according to Japanese practice guidelines for HCC^34,35^.

### Liver function assessment

We assessed liver function using the Child–Pugh classification system^14^ and albumin–bilirubin (ALBI) score^15^. For more detailed evaluation of hepatic function, we used the mALBI grade, which was calculated by subdividing ALBI grade 2 into 2a and 2b^16^.

### Lenvatinib treatment

Lenvatinib (LENVIMA; Eisai, Tokyo, Japan) treatment was started after written informed consent was obtained from each patient. The dose of oral lenvatinib was 8 mg/day in patients who weighed < 60 kg and 12 mg/day in those who weighed ≥ 60 kg. However, the initial dose of lenvatinib was reduced at the discretion of the physician in patients with any of the following: non–Child–Pugh A disease; advanced age; low body weight; pleural effusion, ascites, or gastrointestinal varices at risk of bleeding.

Lenvatinib was discontinued when any unacceptable or serious adverse event or clinical tumor progression occurred. According to the drug manufacturer’s guidelines, the lenvatinib dose was reduced or treatment was interrupted when a patient occurred any grade ≥ 3^36^ severe adverse events, if any intolerable treatment-related adverse events developed, or if there was clinical evidence of HCC progression. In cases of treatment-related adverse events, dosing was reduced or temporarily interrupted until symptoms diminished to grade 1 or 2, according to the manufacturer’s guidelines.

### Evaluation of therapeutic response

Local physicians at each institution evaluated tumors using enhanced computed tomography or magnetic resonance imaging results obtained at 4 or 12 weeks after introducing lenvatinib, in accordance with the modified Response Evaluation Criteria in Solid Tumors (RECIST) guidelines^37,38^.

### Statistical analysis

Continuous variables are expressed as medians (interquartile range). The chi-square test was used for categorical variables. Actuarial analysis of cumulative survival was performed using the Kaplan–Meier method, and differences were tested using the log-rank test with Bonferroni correction. Univariate and multivariate Cox proportional hazards models were used to calculate hazard ratios (HRs) for survival. The concordance (C)-index was used to determine the predictability of survival. We performed multivariate analysis using the following covariates that were previously reported to be risk factors for HCC or predictors of hepatic prognosis: age, sex, Eastern Cooperative Oncology Group performance status (ECOG-PS), HCC etiology, α-fetoprotein, HCC stage, and mALBI grade^4,13,39,40^. We used cut-off values for clinical data as defined in previous reports regarding the risk or prognosis of patients with HCC^4,13,39,40^. In this study, we analyzed clinical data obtained at the start of follow-up. Time-dependent ROC curves for overall survival were obtained with the Kaplan–Meier method using the Child–Pugh score and ALBI score. We calculated the sensitivity and specificity at each survival time using the maximum Youden index (sensitivity + specificity − 1) as the cut-off value^41,42^.

Statistical significance was defined as *p* < 0.05. Statistical analyses were performed with EZR Ver. 1.53 (Saitama Medical Center, Jichi Medical University, Saitama, Japan), which is a graphical user interface for R (The R Foundation for Statistical Computing, Vienna, Austria)^43^. More precisely, it is a modified version of the R commander designed to add statistical functions frequently used in biostatistics.

### Ethics approval

The protocol used in the present study was approved by the Institutional Ethics Committee of Ehime Prefectural Central Hospital (IRB No. 30-66), based on the Guidelines for Clinical Research issued by the Ministry of Health and Welfare of Japan. All methods were carried out in accordance with relevant guidelines and regulations.

### Consent to participate

Written informed consent was obtained from each patient.

## Supplementary Information

Supplementary Figure 1.

Supplementary Figure 2.

Supplementary Table 1.

Supplementary Table 2.

Supplementary Table 3.

Supplementary Table 4.

Supplementary Table 5.

Supplementary Table 6.

Supplementary Table 7.

Supplementary Table 8.

## Data Availability

The datasets are available from the corresponding author on reasonable request.
