# Supplementary material for: Impact of modified albumin–bilirubin grade on survival in patients with HCC who received lenvatinib
Source: Sci Rep. 2021 Jul 14;11:14474. doi: 10.1038/s41598-021-93794-5 (PMC8280227; doi:10.1038/s41598-021-93794-5)
Supplement: Supplementary file 2 — Supplementary Figure 2. [file 41598_2021_93794_MOESM2_ESM.pdf]

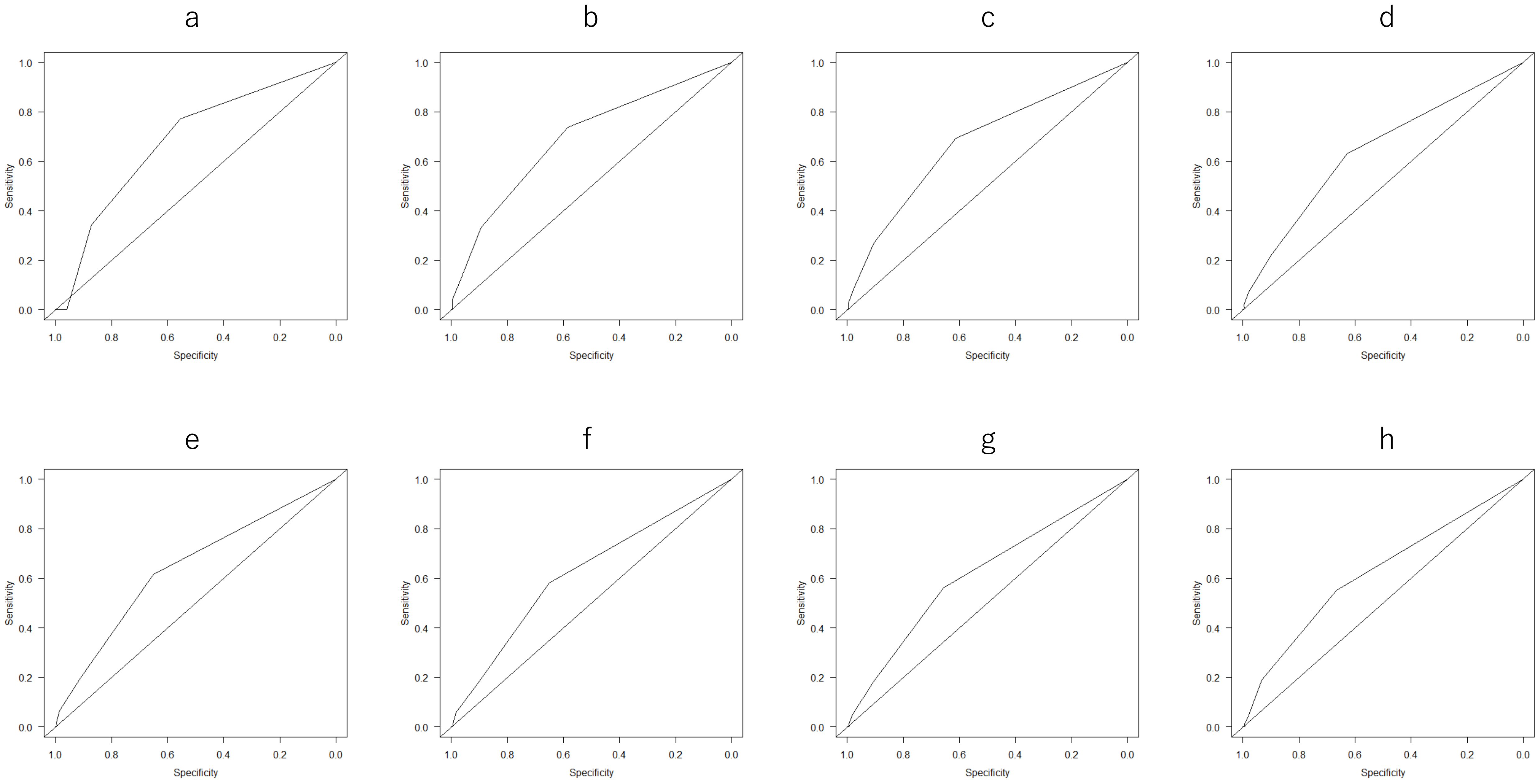

Supplementary figure 2: Time-dependent ROC curves of Child–Pugh scores for overall survival after the start of follow-up
a, 3-month; b, 6-month; c, 9-month; d, 12-month; e, 15-month; f, 18-month; g, 21-month, and h, 24-month.
ROC, receiver operating characteristic.
