# Supplementary material for: Impact of modified albumin–bilirubin grade on survival in patients with HCC who received lenvatinib
Source: Sci Rep. 2021 Jul 14;11:14474. doi: 10.1038/s41598-021-93794-5 (PMC8280227; doi:10.1038/s41598-021-93794-5)
Supplement: Supplementary file 3 — Supplementary Table 1. [file 41598_2021_93794_MOESM3_ESM.pdf]

Supplementary table 1. Multivariate analysis for overall survival

	HR	95% CI	p value
**Age (years)**			
<75 (n=299)	1		0.427
≥75 (n=225)	1.107	0.862–1.421	
**Sex**			
Female (n=126)	1		0.882
Male (n=398)	1.022	0.766–1.364	
**ECOG-PS**			
0 (n=420)	1		0.610
≥1 (n=104)	1.090	0.782–1.521	
**Etiology of HCC**			
Viral (n=291)	1		0.100
Non-viral (n=233)	0.811	0.632–1.041	
**α-fetoprotein (ng/mL)**			
<400 (n=373)	1		0.088
≥400 (n=150)	1.256	0.967–1.631	
**mALBI grade**			
1/2a (n=296)	1		<0.001
2b/3 (n=228)	2.399	1.885–3.054	
**BCLC stage**			
≤B (n=237)	1		0.601
≥C (n=287)	1.412	1.066–1.871	

HR, hazard ratio; CI, confidence interval ECOG-PS, Eastern Cooperative Oncology Group performance status; HCC, hepatocellular carcinoma; mALBI, modified albumin–bilirubin; BCLC, Barcelona Clinic Liver Cancer.
