# Supplementary material for: Impact of modified albumin–bilirubin grade on survival in patients with HCC who received lenvatinib
Source: Sci Rep. 2021 Jul 14;11:14474. doi: 10.1038/s41598-021-93794-5 (PMC8280227; doi:10.1038/s41598-021-93794-5)
Supplement: Supplementary file 4 — Supplementary Table 2. [file 41598_2021_93794_MOESM4_ESM.pdf]

Supplementary table 2. Multivariate analysis for overall survival

	HR	95% CI	p value
**Age (years)**			
<75 (n=299)	1		0.529
≥75 (n=255)	1.084	0.844–1.391	
**Sex**			
Female (n=126)	1		0.946
Male (n=398)	0.990	0.742–1.322	
**ECOG-PS**			
0 (n=420)	1		0.450
≥1 (n=104)	1.138	0.814–1.592	
**Etiology of HCC**			
Viral (n=291)	1		0.111
Non-viral (n=233)	0.817	0.637–1.048	
**α-fetoprotein (ng/mL)**			
<400 (n=373)	1		0.070
≥400 (n=150)	1.273	0.980–1.654	
**Child–Pugh class**			
A (n=448)	1		<0.001
B/C (n=76)	2.048	1.490–2.813	
**BCLC stage**			
≤B (n=237)	1		0.018
≥C (n=287)	1.402	1.059–1.856	

HR, hazard ratio; CI, confidence interval ECOG-PS, Eastern Cooperative Oncology Group performance status; HCC, hepatocellular carcinoma; BCLC, Barcelona Clinic Liver Cancer.
