# Supplementary material for: Impact of modified albumin–bilirubin grade on survival in patients with HCC who received lenvatinib
Source: Sci Rep. 2021 Jul 14;11:14474. doi: 10.1038/s41598-021-93794-5 (PMC8280227; doi:10.1038/s41598-021-93794-5)
Supplement: Supplementary file 5 — Supplementary Table 3. [file 41598_2021_93794_MOESM5_ESM.pdf]

Supplementary table 3. Therapeutic response according to the mALBI grade

	mALBI grade		p-value
	1/2a (n=296)	2b/3 (n=228)	
CR	12 (4.3%)	9 (4.5%)	0.102
PR	103 (36.5%)	55 (27.8%)	
SD	119 (42.2%)	85 (42.9%)	
PD	48 (17.0%)	49 (24.7%)	
Not evaluated	14	30	
ORR	40.8%	32.3 %	0.069
DCR	83.0%	75.3%	0.049

mALBI, modified albumin–bilirubin**;** CR, complete response; PR, partial response; SD, stable disease PD, progression disease; ORR, overall response rate; DCR, disease control rate.
