# Supplementary material for: Impact of modified albumin–bilirubin grade on survival in patients with HCC who received lenvatinib
Source: Sci Rep. 2021 Jul 14;11:14474. doi: 10.1038/s41598-021-93794-5 (PMC8280227; doi:10.1038/s41598-021-93794-5)
Supplement: Supplementary file 6 — Supplementary Table 4. [file 41598_2021_93794_MOESM6_ESM.pdf]

Supplementary table 4. Therapeutic response according to the Child–Pugh class

	Child–Pugh class		p-value
	A (n=448)	B/C (n=76)	
CR	20 (4.8%)	1 (1.5%)	0.003
PR	140 (33.8%)	18 (27.3%)	
SD	182 (44.0%)	22 (33.3%)	
PD	72 (17.4%)	25 (37.9%)	
Not evaluated	34	10	
ORR	38.6%	28.8 %	0.134
DCR	82.6%	62.1%	<0.001

CR, complete response; PR, partial response; SD, stable disease PD, progression disease; ORR, overall response rate; DCR, disease control rate.
