# Supplementary material for: Impact of modified albumin–bilirubin grade on survival in patients with HCC who received lenvatinib
Source: Sci Rep. 2021 Jul 14;11:14474. doi: 10.1038/s41598-021-93794-5 (PMC8280227; doi:10.1038/s41598-021-93794-5)
Supplement: Supplementary file 7 — Supplementary Table 5. [file 41598_2021_93794_MOESM7_ESM.pdf]

Supplementary table 5. Adverse events according to the mALBI grade

	mALBI grade 1/2a (n=296)	mALBI grade 2b/3 (n=228)	p-value
Palmar-plantar erythrodysesthesia			
Any grade	94 (31.8%)	42 (18.4%)	0.001
Grade ≥3	7 (2.4%)	6 (2.6%)	1.000
Fatigue			
Any grade	101 (34.1%)	73 (32.0%)	0.641
Grade ≥3	20 (6.8%)	9 (3.9%)	0.182
Decreased appetite			
Any grade	93 (31.4%)	79 (34.6%)	0.454
Grade ≥3	15 (5.1%)	19 (8.3%)	0.153
Proteinuria			
Any grade	89 (30.1%)	41 (18.0%)	0.002
Grade ≥3	25 (8.4%)	9 (3.9%)	0.048
Diarrhea			
Any grade	64 (21.6%)	33 (14.5%)	0.041
Grade ≥3	10 (3.4%)	3 (1.3%)	0.163
Hypothyroidism			
Any grade	89 (30.1%)	47 (20.6%)	0.016
Grade ≥3	4 (1.4%)	3 (1.3%)	1.000
Hypertension			
Any grade	80 (27.0%)	35 (15.4%)	0.001
Grade ≥3	7 (2.4%)	6 (2.6%)	1.000
Other			
Any grade	78 (26.4%)	64 (28.1%)	0.372
Grade ≥3	20 (6.8%)	21 (9.2%)	0.692

mALBI, modified albumin–bilirubin.
