# Supplementary material for: Impact of modified albumin–bilirubin grade on survival in patients with HCC who received lenvatinib
Source: Sci Rep. 2021 Jul 14;11:14474. doi: 10.1038/s41598-021-93794-5 (PMC8280227; doi:10.1038/s41598-021-93794-5)
Supplement: Supplementary file 8 — Supplementary Table 6. [file 41598_2021_93794_MOESM8_ESM.pdf]

Supplementary table 6. Adverse events according to the Child–Pugh class

	Child–Pugh class A (n=448)	Child–Pugh class B/C (n=76)	p-value
Palmar-plantar erythrodysesthesia			
Any grade	125 (27.9%)	11 (14.5%)	0.016
Grade ≥3	13 (2.9%)	0 (0.0%)	0.232
Fatigue			
Any grade	148 (33.0%)	26 (34.2%)	0.895
Grade ≥3	25 (5.6%)	4 (5.3%)	1.000
Decreased appetite			
Any grade	148 (33.0%)	24 (31.6%)	0.895
Grade ≥3	30 (6.7%)	4 (5.3%)	0.804
Proteinuria			
Any grade	113 (25.2%)	17 (22.4%)	0.668
Grade ≥3	29 (6.5%)	5 (6.6%)	1.000
Diarrhea			
Any grade	88 (19.6%)	9 (11.8%)	0.113
Grade ≥3	13 (2.9%)	0 (0.0%)	0.232
Hypothyroidism			
Any grade	119 (26.6%)	17 (22.4%)	0.269
Grade ≥3	5 (1.1%)	2 (2.6%)	0.482
Hypertension			
Any grade	110 (24.6%)	5 (6.6%)	<0.001
Grade ≥3	13 (2.9%)	0 (0.0%)	0.232
Other			
Any grade	126 (28.1%)	16 (21.1%)	0.213
Grade ≥3	38 (8.5%)	3 (3.9%)	0.247
