# Supplementary material for: Impact of modified albumin–bilirubin grade on survival in patients with HCC who received lenvatinib
Source: Sci Rep. 2021 Jul 14;11:14474. doi: 10.1038/s41598-021-93794-5 (PMC8280227; doi:10.1038/s41598-021-93794-5)
Supplement: Supplementary file 9 — Supplementary Table 7. [file 41598_2021_93794_MOESM9_ESM.pdf]

Supplementary table 7. Multivariate analysis for overall survival according to the adverse events and mALBI grade

	HR	95% CI	p value
**Palmar-plantar erythrodysesthesia**			
No (n=388)	1		0.008
Yes (n=136)	0.679	0.511–0.903	
**Fatigue**			
No (n=350)	1		0.636
Yes (n=174)	0.934	0.705–1.238	
**Decreased appetite**			
No (n=352)	1		0.109
Yes (n=172)	1.252	0.951–1.647	
**Proteinuria**			
No (n=394)	1		0.008
Yes (n=130)	0.646	0.467–0.892	
**Hypothyroidism**			
No (n=388)	1		0.305
Yes (n=136)	0.854	0.631–1.155	
**Hypertension**			
No (n=409)	1		0.586
Yes (n=115)	0.919	0.677–1.247	
**mALBI grade**			
1/2a (n=296)	1		<0.001
2b/3 (n=228)	2.234	1.747–2.857	

HR, hazard ratio; CI, confidence interval; mALBI, modified albumin–bilirubin.
