# Supplementary material for: Impact of modified albumin–bilirubin grade on survival in patients with HCC who received lenvatinib
Source: Sci Rep. 2021 Jul 14;11:14474. doi: 10.1038/s41598-021-93794-5 (PMC8280227; doi:10.1038/s41598-021-93794-5)
Supplement: Supplementary file 10 — Supplementary Table 8. [file 41598_2021_93794_MOESM10_ESM.pdf]

**Supplementary table 8. Multivariate analysis for overall survival according to the adverse events and Child–Pugh class**

| | HR | 95% CI | p value |
|---|---|---|---|
| **Palmar-plantar erythrodysesthesia** | | | |
| No (n=388) | 1 | | 0.003 |
| Yes (n=136) | 0.648 | 0.487–0.862 | |
| **Fatigue** | | | |
| No (n=350) | 1 | | 0.418 |
| Yes (n=174) | 0.889 | 0.668–1.182 | |
| **Decreased appetite** | | | |
| No (n=352) | 1 | | 0.068 |
| Yes (n=172) | 1.295 | 0.981–1.710 | |
| **Proteinuria** | | | |
| No (n=394) | 1 | | 0.001 |
| Yes (n=130) | 0.584 | 0.421–0.808 | |
| **Hypothyroidism** | | | |
| No (n=388) | 1 | | 0.313 |
| Yes (n=136) | 0.856 | 0.632–1.158 | |
| **Hypertension** | | | |
| No (n=409) | 1 | | 0.572 |
| Yes (n=115) | 0.915 | 0.672–1.246 | |
| **Child–Pugh class** | | | |
| A (n=448) | 1 | | <0.001 |
| B/C (n=76) | 2.021 | 1.464–2.790 | |

HR, hazard ratio; CI, confidence interval.
